# Tumor-associated macrophages promote neuroblastoma via STAT3 phosphorylation and up-regulation of c-MYC

**DOI:** 10.18632/oncotarget.21066

**Published:** 2017-09-16

**Authors:** Michael D. Hadjidaniel, Sakunthala Muthugounder, Long T. Hung, Michael A. Sheard, Soheila Shirinbak, Randall Y. Chan, Rie Nakata, Lucia Borriello, Jemily Malvar, Rebekah J. Kennedy, Hiroshi Iwakura, Takashi Akamizu, Richard Sposto, Hiroyuki Shimada, Yves A. DeClerck, Shahab Asgharzadeh

**Affiliations:** ^1^ Children's Hospital Los Angeles, Children's Center for Cancer and Blood Diseases, Division of Hematology, Oncology and Blood & Marrow Transplantation, and The Saban Research Institute, Los Angeles, CA, USA; ^2^ The First Department of Medicine, Wakayama Medical University, Wakayama, Japan; ^3^ Keck School of Medicine, University of Southern California, Los Angeles, CA, USA

**Keywords:** neuroblastoma, tumor-associated macrophages, tumor microenvironment, STAT3, MYC

## Abstract

Tumor-associated macrophages (TAMs) are strongly associated with poor survival in neuroblastomas that lack *MYCN* amplification. To study TAM action in neuroblastomas, we used a novel murine model of spontaneous neuroblastoma lacking *MYCN* amplification, and observed recruitment and polarization of TAMs, which in turn enhanced neuroblastoma proliferation and growth. In both murine and human neuroblastoma cells, we found that TAMs increased STAT3 activation in neuroblastoma cells and transcriptionally up-regulated the *MYC* oncogene. Analysis of human neuroblastoma tumor specimens revealed that MYC up-regulation correlates with markers of TAM infiltration. In an *IL6*^ko^ neuroblastoma model, the absence of IL-6 protein had no effect on tumor development and prevented neither STAT3 activation nor MYC up-regulation. In contrast, inhibition of JAK-STAT activation using AZD1480 or the clinically admissible inhibitor ruxolitinib significantly reduced TAM-mediated growth of neuroblastomas implanted subcutaneously in NOD scid gamma mice. Our results point to a unique mechanism in which TAMs promote tumor cells that lack amplification of an oncogene common to the malignancy by up-regulating transcriptional expression of a distinct oncogene from the same gene family, and underscore the role of IL-6-independent activation of STAT3 in this mechanism. Amplification of *MYCN* or constitutive up-regulation of *MYC* protein is observed in approximately half of high-risk tumors; our findings indicate a novel role of TAMs as inducers of *MYC* expression in neuroblastomas lacking independent oncogene activation.

## INTRODUCTION

The role of inflammation in promoting tumor growth and shaping the immune response in the tumor microenvironment (TME) has emerged as an important theme in cancer biology [1]. Tumor-associated macrophages (TAMs), which most closely resemble M2-polarized macrophages, are major contributors to the TME, and are found in various types of human cancers [2–4]. The presence of TAMs and high levels of specific chemokines and cytokines, including interleukin-6 (IL-6), is associated with lower survival rates in patients with several types of tumors, including neuroblastoma (NBL) for which the prognosis is poor in children with high-risk disease [3, 5–11]. NBLs lacking *MYCN* amplification express CC chemokine ligand 2 (CCL2) also called monocyte chemotactic protein-1 (MCP1), which is known to recruit monocytes and promote their polarization to M2-like TAMs, at least in part through interaction with the receptor CCR2 [12, 13]. The presence of TAMs in NBL tumors correlates with NBL disease stage, with the largest infiltration observed in children with metastatic disease. Expression of inflammation-associated genes, including CD14, CD16, CD33, IL-10, and IL-6R, was shown to contribute to the classification score of children with high-risk disease [6]. This raised the question of whether effective therapeutic approaches in children with NBL could be based on the targeting of inflammation-associated biologic pathways in the TME, for example using an anti-IL-6 monoclonal antibody (mAb) to target the IL-6 pathway as evaluated in adult cancers [14].

IL-6 is considered a mediator of monocyte-mediated proliferation of tumor cells through its binding to the IL-6 receptor (IL-6R). The levels of IL-6 in NBL patient serum and bone marrow have been reported to be elevated [11, 15], and the combination of IL-6 plus soluble IL-6R from monocytes has been shown to activate STAT3 and mediate drug resistance in NBL cells [16]. It has been reported that IL-6 levels in peripheral blood at diagnosis correlate with features of high-risk NBL and poor prognosis [15]. In addition to the complex of IL-6 with the soluble subunit of IL-6R, the membrane-bound common gp130 subunit of IL-6R (CD130) can be activated by other ligand(s) such as ciliary neurotrophic factor and oncostatin M, and activation of the JAK-STAT signaling pathway downstream of IL-6R ligation plays a significant role in the proliferation, survival, invasion and immunosuppressive character of cancers [17–20].

In this study, we focused on the role of TAMs and STAT3 in NBL and its TME. While TAMs are observed in primary tumors of children with high-risk disease, the mechanism of their contribution to tumor growth and the relative role of IL-6 in STAT3 activation are not clearly understood. Here, we elucidate the functional and biologic roles of TAMs using pharmacologic and genetic approaches, corroborated by gene expression data from a large cohort of NBL human tumors. Our findings reveal novel insights about the contributions of TAMs and IL-6 to the regulation of MYC expression, and validate the JAK-STAT inhibitor ruxulitinib as a potential modality for NBL therapy.

## RESULTS

### Tumor-associated macrophages enhance NBL proliferation and growth via MYC up-regulation

To better understand the relationship of tumor growth to proliferation in the context of tumor-TAM interactions, we utilized a previously described transgenic murine NBL model (NB-Tag) [21]. Our extensive characterization of this model by gene expression, DNA copy number, and immunohistochemistry demonstrated that it closely resembles human tumors lacking *MYCN* amplification (Supplemental Figure 1). The NB-Tag tumors were characterized as NBL by tyrosine hydroxylase (TH) immunohistochemistry, and further classified by a pathologist as undifferentiated NBL with high mitosis-karyorrhexis index. NB-Tag tumors lack *MYCN* genomic amplification in the murine chromosome 12 region, which is syntenic to human chromosome 2p24 region that contains human *MYCN*. NB-Tag tumors (n = 3) also clustered more tightly with human NB tumors (n = 102) than any other normal human tissues (n = 131) using principle component analysis (Supplementary Figure 1H).

**Figure 1:**
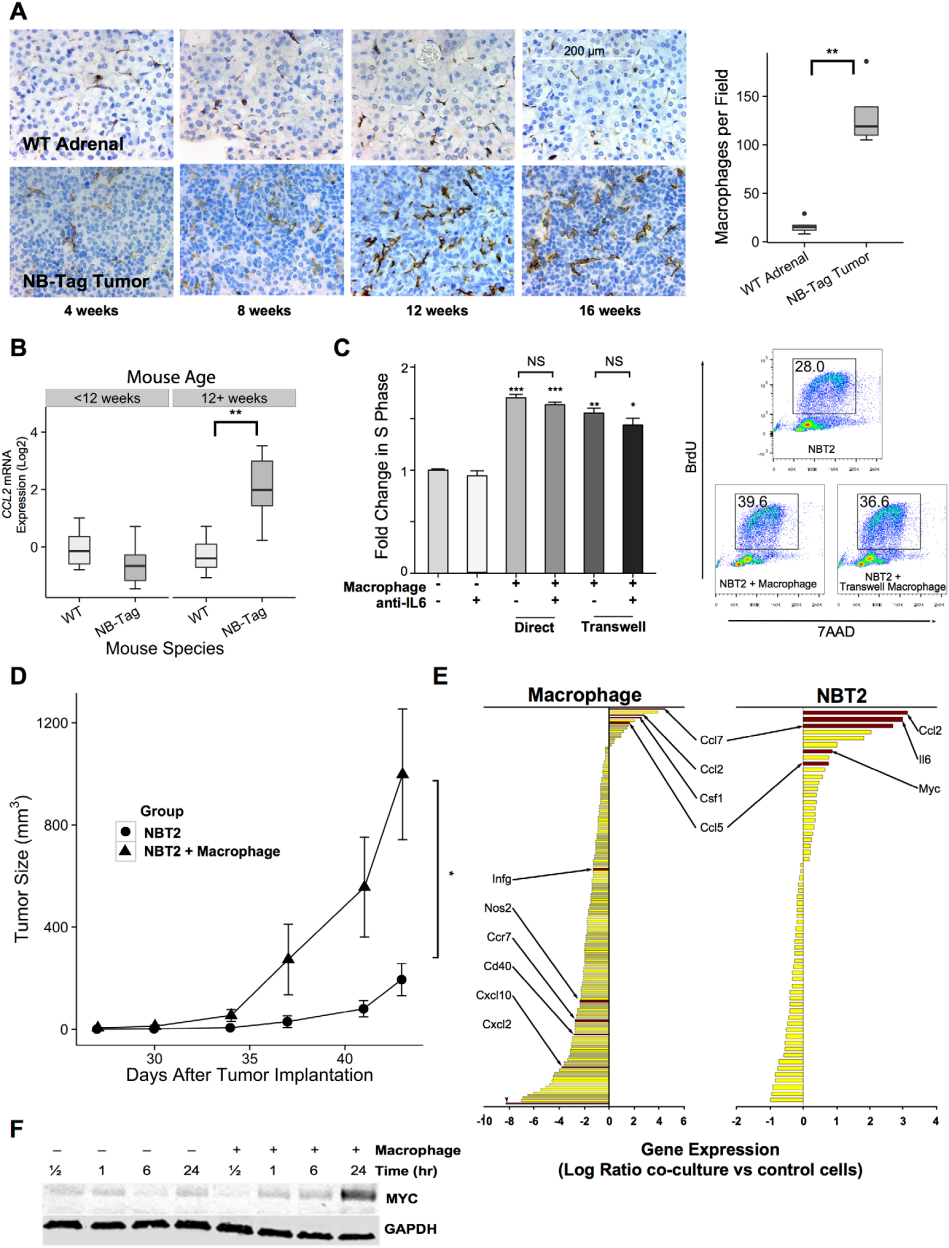
TAM infiltration in NB-Tag tumors is associated with tumor proliferation and induction of MYC expression **(A)** Left: Representative immunohistochemical images of macrophages (detected using anti-F4/80 antibody) in the adrenal medulla of WT mice and in tumors arising from adrenal glands of NB-Tag mice at various ages; Right: Boxplots of macrophage counts (F4/80^+^ cells) from IHC images of mice >12 weeks of age compared to age-matched adrenal medulla. (At least 5 fields from each tumor or adrenal gland section per specimen and 4-6 specimens per group were analyzed) (** p <0.005); **(B)** Boxplot of CCL2 gene expression levels measured by RT-PCR comparing NB-Tag tumors versus WT adrenal glands in mice < 12 weeks of age and > 12 weeks of age (n = 6 per group) (** p < 0.005); **(C)** Left: Mean fold change (+SD) in S phase frequency, as measured by BrDU incorporation, of NBT2 cells cultured with and without macrophages in direct contact or in transwell plates and in the presence or absence of anti-IL-6 neutralizing antibody (10 μg/ml). Macrophages were gated out of analyses according to staining for CD45. Data were compiled from at least 3 independent experiments in triplicates (* p < 0.05, ** p < 0.005, *** p < 0.0005); Right: Representative flow cytometry profile of incorporation of BrdU in NBT2 cells as measured in various experimental designs; **(D)** Tumor growth in NSG mice injected with 1x10^0^ NBT2 tumor cells alone in one shoulder or co-injected with equal numbers of macrophages in the opposite shoulder (macrophages were conditioned for 24-36 hours with NBT2 cells in the transwell system) (n = 4 mice per group, * ANOVA p = 0.03); **(E)** Average fold change in gene expression levels (using Nanostring Mouse Inflammation Kit) of tumor-conditioned macrophages compared to peritoneal macrophages (left panel) and of NBT2 cells cultured with macrophages compared to naïve NBT2 cells (right panel) (data obtained from three independent experiments); **(F)** Expression levels of MYC protein in lysates of NBT2 cells cultured in transwells with or without macrophages as analyzed by immunoblotting.

The reproducible growth characteristics of NB-Tag tumors (Supplementary Figure 1A, 1F) allowed us to examine the development of the TME. We observed significant increase in infiltration of macrophages in NB-Tag tumors over time compared to control adrenal glands (Figure 1A). The macrophage infiltration was prominent around 12-13 weeks of age, prior to the tumors becoming visible by magnetic resonance imaging (MRI) (Figure 1A and Supplementary Figure 1A), and coincided with an increase in *CCL2* mRNA expression in tumors of mice aged > 12 weeks (Figure 1B). We have previously observed that high *CCL2* mRNA expression in humans occurs almost exclusively in *MYCN* non-amplified NBL tumors [12]; thus, high expression of *CCL2* by NB-Tag tumors further supports that this model is a *bona fide* murine model of human tumors lacking *MYCN* amplification.

To investigate the effects of macrophages on NBL cell proliferation and growth, we co-cultured a NB-Tag-derived mouse cell line (NBT2) with MACS-purified peritoneal-derived mouse macrophages, and measured S-phase changes by BrdU labeling and flow cytometry. A significant increase in tumor cell proliferation by up to 70% over the basal rate was observed when cells were in direct contact and by up to 55% when using the transwell system (p < 0.0005 and p < 0.005 respectively, Figure 1C). Peritoneal macrophages conditioned *in vitro* by NBT2 cells for 24 hours prior to subcutaneous co-injection with tumor cells in syngeneic C57B6 mice also significantly enhanced subcutaneous tumor growth compared to NBT2 tumor cells injected alone (p < 0.001, Figure 1D).

Gene expression analyses of macrophages prior to and after co-cultures with NBT2 cells (Figure 1E) revealed that expression of *CXCL10*, *CCR7*, and *NOS2* (*iNOS*), which are known to be associated with M1 polarization, was down-regulated, while expression of *CCL2*, *CCL7* (another member of the MCP family) and *CCL5*, known to be expressed by TAMs, was up-regulated [13, 22]. Expression of *CCL2* also increased in NBT2 cells after co-cultures, suggesting autocrine and paracrine activation of the CCL2-CCR2 axis for further recruitment and polarization of macrophages to the TAM phenotype [13]. Interestingly, we conjointly observed an increase in the expression of the *MYC* gene (*c-MYC*) in NBT2 cells after co-cultures. The up-regulation of *MYC* mRNA was associated with a similar increase in the level of protein expression after 24 hours of co-culture with peritoneal macrophages (Figure 1F).

**Figure 2:**
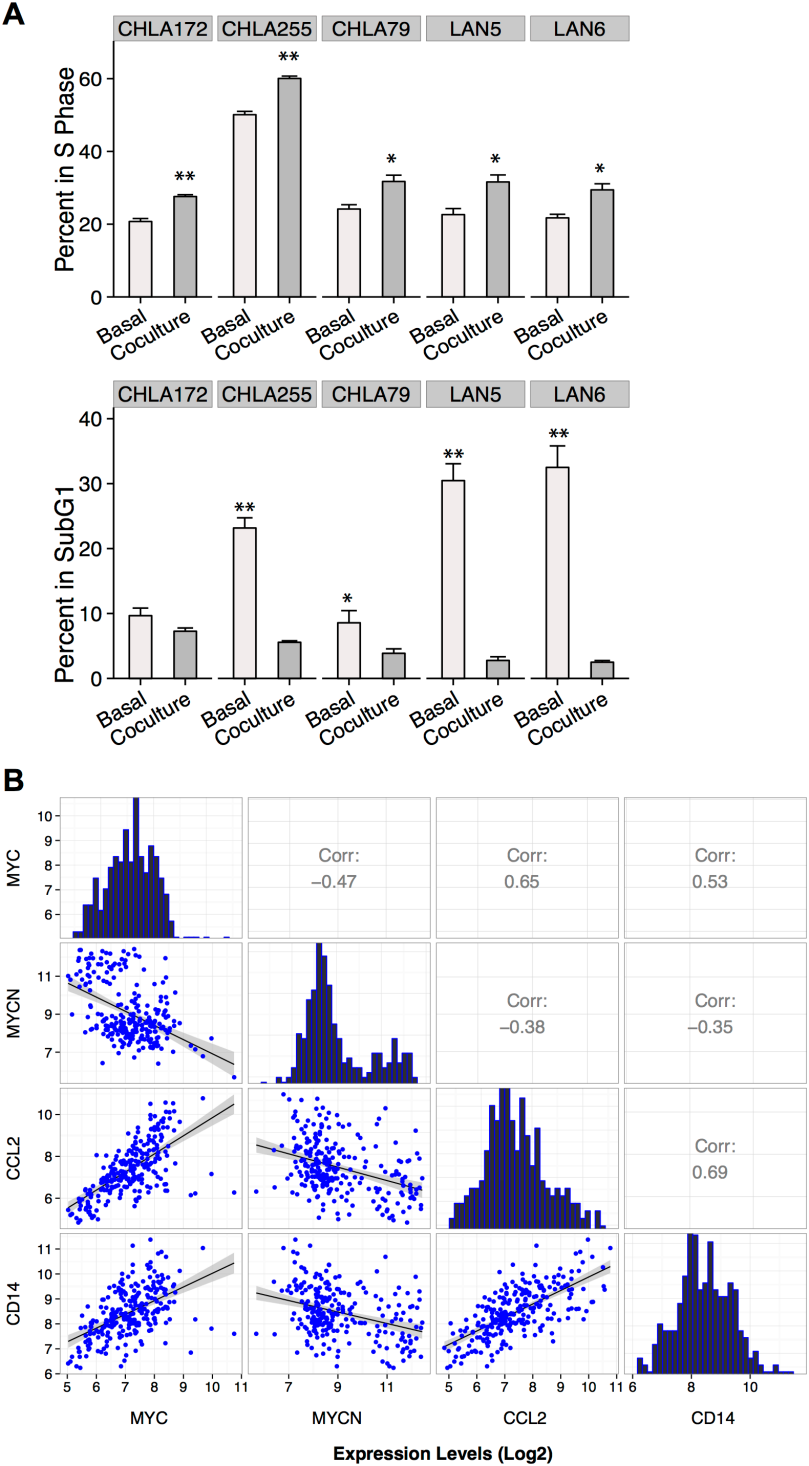
MYC up-regulation in human NBL associated with presence of macrophages **(A)** Mean percentage (+SD) of BrdU and 7AAD (sub-G1 phase) positive human NBL cells cultured in the presence or absence of macrophages polarized to M2 (1:1 ratio) by co-culturing with NBT2 cells followed by MACS purification. Data were compiled from at least 3 independent experiments performed in triplicates (* *p* <0.05, ** *p* <0.005); **(B)** Pearson correlation analysis of *MYC*, *MYCN*, *CCL2*, and *CD14* expression levels (log2 base) based on microarray data from 249 primary human NBL tumors.

The proliferative effect of macrophages on NBL cells was also observed in co-cultures of human macrophages with human cell lines (Figure 2A). Five of five human cell lines showed a significant increase in S-phase (range 1.2-1.5 fold increase, p<0.05) and a significant decrease in sub-G1 phase (average decrease of 10 fold, p<0.0005) when co-cultured with macrophages polarized to M2 phenotype (1:1 ratio). A 1.5- to 2-fold increase in MYC protein expression by immunoblotting was also observed among human cell lines lacking *MYCN* amplification (LAN-6, CHLA-172, and CHLA-79; Figure 5C); however, this increase in MYC protein levels was not observed in CHLA-255 or LAN-5, as these cell lines already overexpress MYC and MYCN proteins, respectively, at baseline (data not shown).

**Figure 3:**
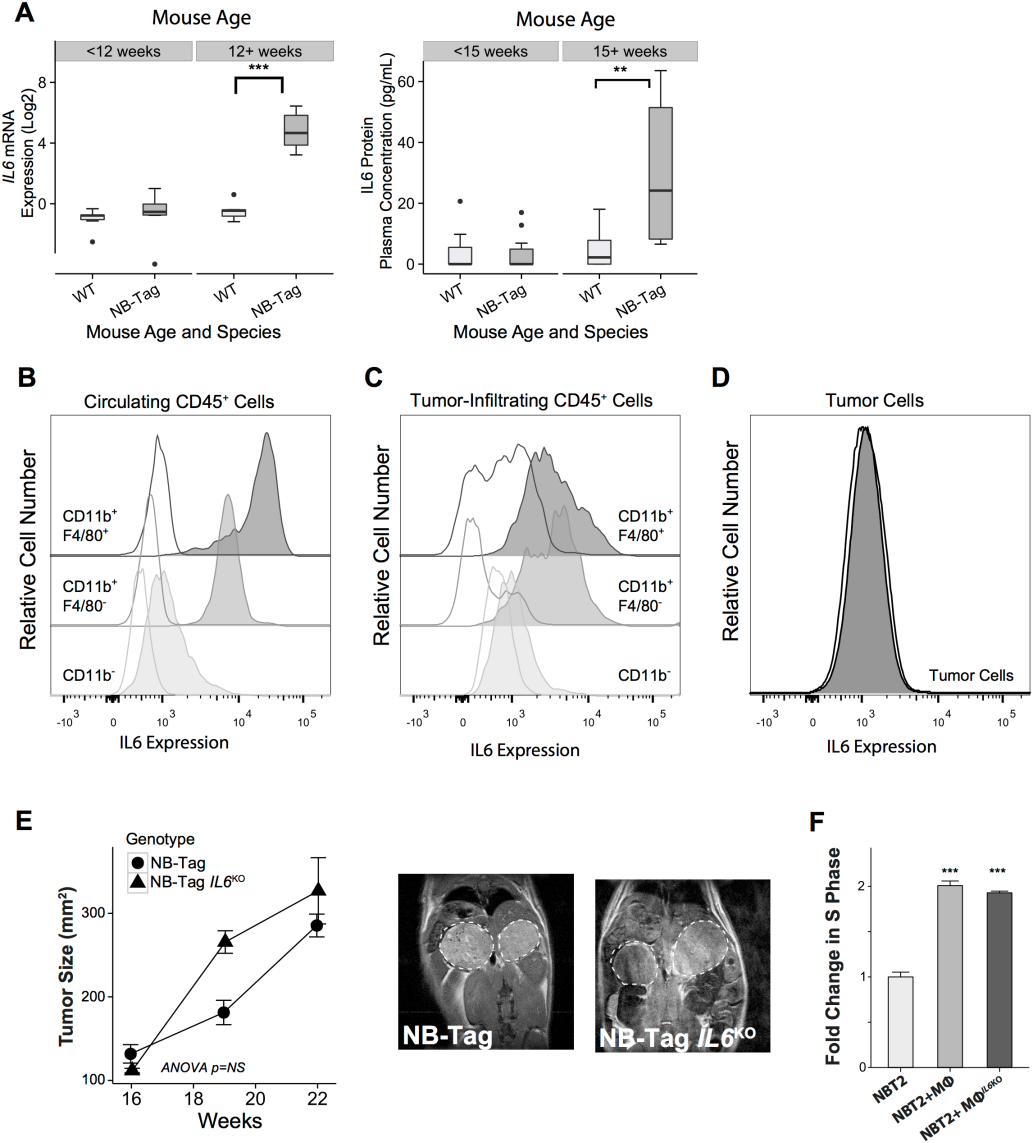
Macrophage-induced NBL proliferation is unaffected by absence of IL-6 **(A)** Left: Boxplot representing the distribution of *IL6* mRNA expression levels comparing NB-Tag tumors versus WT adrenal glands in mice <12 weeks of age and >12 weeks of age (n=6 per group);Right**:** Plasma IL-6 protein concentrations, as measured by the Luminex assay, comparing plasma obtained from NB-Tag mice with clearly visible tumors by MRI (≥ 15 weeks of age) to NB-Tag < 15 weeks of age and their age-matched wild-type controls (WT n = 19, NB-Tag n = 20); **(B-D)** Representativeflow cytometry dot plots illustrating intracellular IL-6 protein expression in circulating and tumor infiltrating CD45^+^ immune cell subsets, and tumor cells (CD45^-^) in a 24-week-old NB-Tag mouse. Clear histograms represent isotype control antibodies. Monocytic lineage cells (CD11b^+^) are separated into F4/80 positive and negative populations; **(E)** Mean tumor size (+SD) of NB-Tag (n = 5) and NB-Tag-*IL6*^KO^ (n = 3) as measured by MRI over time (ANOVA p=NS), and representative MRI images (16-week old mice); **(F)** Mean fold change (+SD) in BrdU-positive NBT2 cells cultured in the presence of WT macrophages (Mϕ) or *IL6*^KO^ macrophages compared to control NBT2 cells (*** *p* < 0.0005).

**Figure 4:**
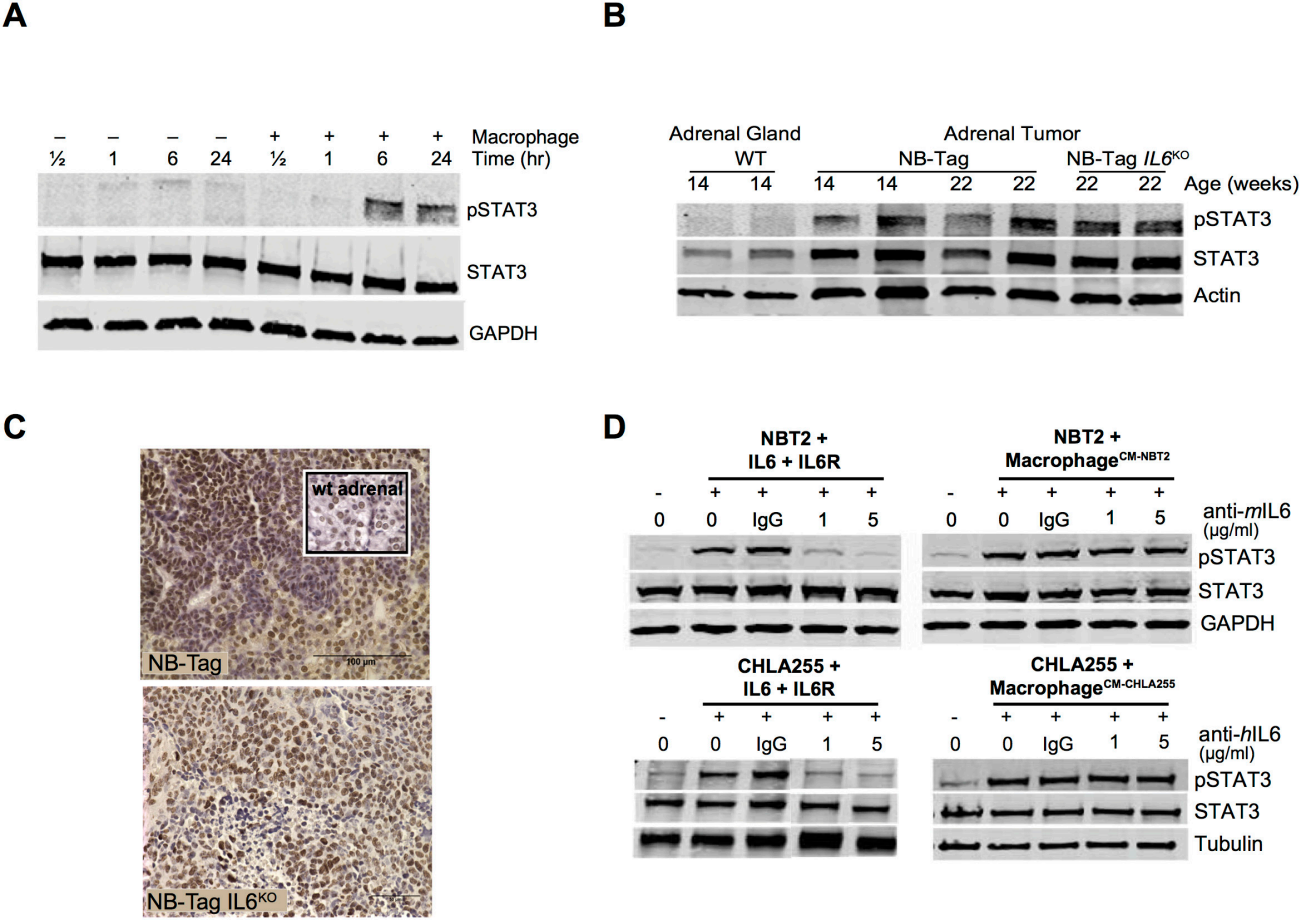
STAT3 activation in human and murine NBL cells by macrophages does not require IL-6 **(A)** Immunoblot analysis showing STAT3 expression and phosphorylated STAT3 (pSTAT3) levels over time in protein lysates of NBT2 cells cultured in transwells with and without syngeneic murine macrophages. GAPDH is used as control for protein loading; **(B)** STAT3 expression and pSTAT3 levels assessed by immunoblotting protein lysates from adrenal glands of WT, NB-Tag, and NB-Tag-*IL6*^KO^ mice (14-22 weeks of age); **(C)** Representative images of pSTAT3 IHC in tumors of NB-Tag and NB-Tag-*IL6*^KO^ mice (inset: WT adrenal gland); **(D)** Immunoblots of STAT3 and pSTAT3 levels in NBT2 (murine) and CHLA-255 (human) NBL cells at basal level, and in the presence of IL-6 (10 ng/ml) or sIL-6R (25 ng/ml) either alone or with macrophages previously conditioned with tumor cell media, and incubated with IgG (control) or species-specific neutralizing anti-IL-6 mAb (1 and 5 μg/ml).

**Figure 5:**
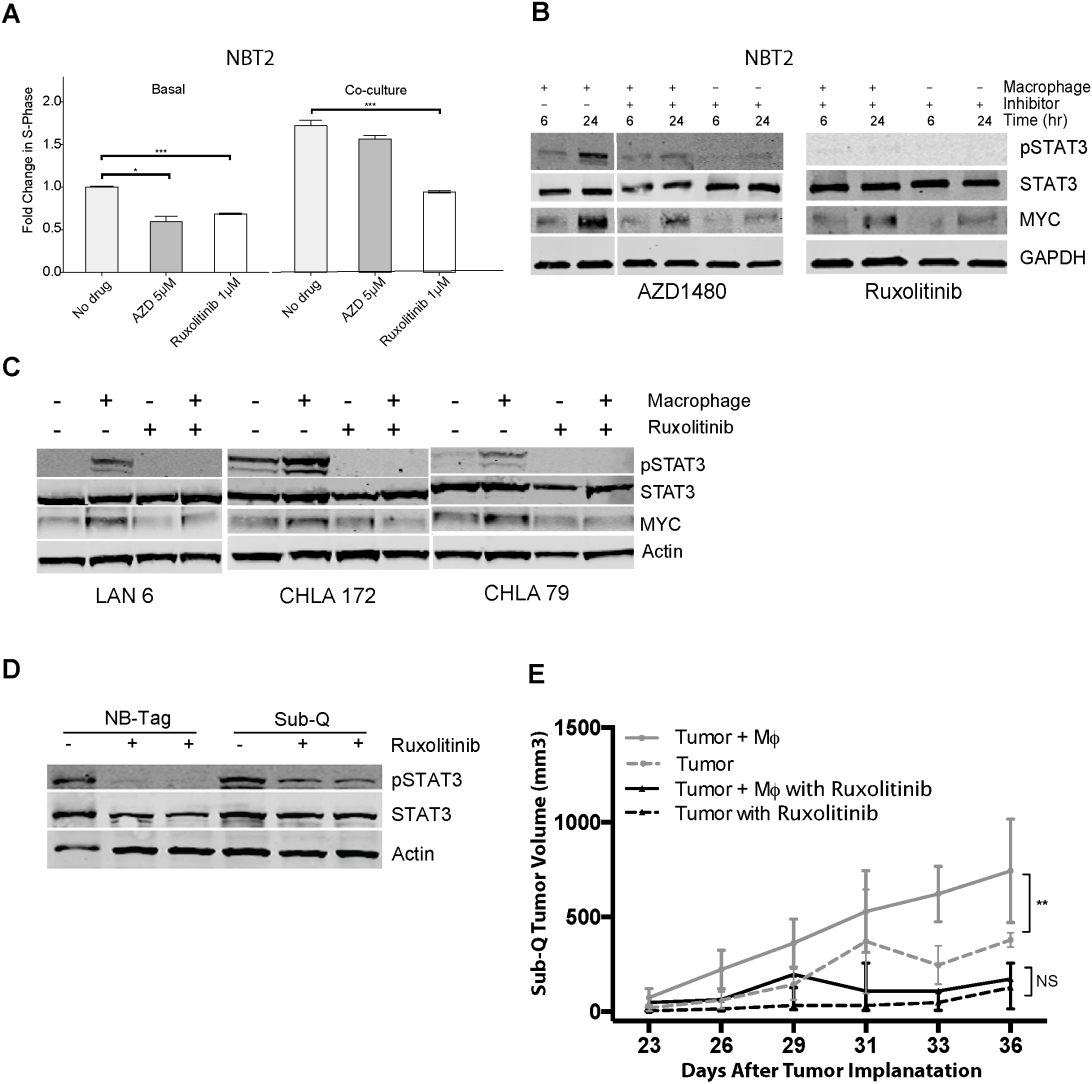
Macrophage-mediated proliferation of NBT2 cells is dependent on STAT3 phosphorylation **(A)** Mean fold change in percent of BrdU-positive NBT2 cells treated with solvent or JAK1/2 inhibitors AZD1480 (AZD) or ruxolitinib at indicated concentrations for NBT2 cells either alone or in co-culture with macrophages. Data were compiled from at least 3 independent experiments in triplicates; **(B)** Effects of AZD1480 and ruxolinitib on pSTAT3, STAT3, and MYC protein levels in lysates of NBT2 cells without or with a 6 and 24 hour co-culture with syngeneic macrophages in presence of AZD1480 (5 μM) or ruxolitinib (1 μM) as assessed by immunoblotting; **(C)** Immunoblot analysis of pSTAT3, STAT3, and MYC levels in protein lysates of three low MYC-expressing human NBL cell lines (LAN-6, CHLA-172, and CHLA-79) after 24 hour co-cultures with macrophages without or with ruxolitinib (1 μM); **(D)** Immunoblot analysis of STAT3 and pSTAT3 levels in protein lysates of tumors from NB-Tag mice or of subcutaneous tumors (Sub-Q) growing in NSG mice treated with ruxolitinib (60 mg/kg) by oral gavage twice daily for one week. **(E)** Tumor growth in NSG mice injected subcutaneously with 1X10^6^ NBT2 tumor cells alone in one shoulder or co-injected in the opposite shoulder with equal number of macrophages that were conditioned for 24-36 hours with NBT2 cells in the transwell system. Animals were administered ruxolitinib (60 mg/kg) or drug vehicle by oral gavage twice daily for 3 weeks [ANOVA p < 0.005 between the untreated group (n = 12) and the treated group (n = 6); no significant difference was observed between treatment groups].

We next analyzed the expression profiles of 249 primary human NBL tumor samples from the TARGET project to assess a potential association between *MYC* expression, *MYCN* expression, and the CCL2-CCR2 axis. We observed mutually exclusive expression of *MYC* and *MYCN* as well as negative correlation of *MYCN* with *CCL2*, which corroborates previous findings by our group (Figure 2B) [23]. Importantly, we found a highly positive correlation between *MYC* and both *CCL2* and *CD14* (0.65 and 0.53, respectively, p < 0.01), while a negative correlation was obtained with *MYCN* expression (-0.38, and -0.35, respectively, p < 0.01; Figure 2B). Overall, our data suggest that NBL tumor cells lacking *MYCN* amplification recruit and polarize macrophages to an M2-like phenotype, which in turn enhance NBL proliferation and growth through up-regulation of the MYC protein.

### NBL-macrophage co-culture is associated with IL-6 expression, but NBL proliferation and growth do not require IL-6

IL-6 and IL-6R have been shown to promote tumor growth, and we previously identified the *IL6R* gene as one of the inflammation-related genes in a 14-gene prognostic signature in children with high-risk tumors lacking *MYCN* amplification [6]. We analyzed the expression level of *IL6* mRNA and IL-6 protein over time in tumor samples and plasma from NB-Tag mice, and noted that its increase correlated with the time of highest macrophage infiltration in the tumors, *i.e*., > 12 weeks of age (Figure 1A and Figure 3A). In order to identify the cells responsible for *IL6* gene expression in the NB-Tag model, we analyzed the intracellular levels of IL-6 protein by flow cytometry in circulating immune cells and single cells dissociated from tumors. The tumor-infiltrating myeloid cells (CD11b^+^F4/80^+^ and CD11b^+^/F4/80^-^) and circulating monocytes (CD11b^+^) in blood were identified as the predominant cells producing IL-6 (Figure 3B, 3C). Minimal IL-6 production was observed in tumor cells (CD45^-^ cells) or non-myeloid (CD11^-^) immune cells (Figure 3B, 3C).

Considering the well-known effect of IL-6 on STAT3 activation and the presence of IL-6 in our tumor model, we next asked whether IL-6 produced by TAMs contributes to the proliferative advantage provided by macrophages in our co-culture system. To our surprise, neutralizing IL-6 in the co-culture media with maximal levels of anti-IL-6 antibodies did not significantly decrease proliferation of NBT2 cells (3.5% and 7.3% decrease in BrdU positive cells in direct and transwell systems; *p=NS*, respectively) (Figure 1C). To definitively assess the impact of IL-6 on NB-Tag tumor development, we generated *IL6*-deficient double transgenic mice (NB-Tag/*IL6*^KO^), and studied their tumor growth kinetics. Supporting our co-culture findings, there was no difference in growth patterns between the NB-Tag/*IL6*^KO^ and NB-Tag mice (Figure 3E). Furthermore, peritoneal macrophages obtained from the NB-Tag/*IL6*^KO^ mice replicated the growth-promoting effects on NBT2 cells *in vitro* just as well as their wild-type counterpart (Figure 3F) despite their inability to produce IL-6 protein (Supplementary Figure 2). These findings indicate that IL-6 is sufficient but not required for macrophage-mediated promotion of NBL.

### Macrophages can induce pSTAT3 in NBL independently of IL-6

As IL-6 is known to activate STAT3 in the TME, we evaluated STAT3 activation (assessed by phosphorylation of STAT3) in co-cultures of NBT2 cells and macrophages. A strong increase in phosphorylation of STAT3 (pSTAT3) was observed in NBT2 cells cultured with NB-Tag macrophages starting after 6 hours of co-culture (Figure 4A), *i.e*., well before the observed increase in MYC protein levels (Figure 1F). STAT3 was also similarly activated in IL-6-deficient and IL-6-producing tumors as assessed by immunoblotting and immunohistochemistry (IHC) (Figure 4B, 4C).

To verify that IL-6R was functional in NBT2 cells, we tested the activation of STAT3 in cells treated with exogenous IL-6 in the presence of sIL-6R, the mediator of IL-6 *trans* signaling. These experiments demonstrated that pSTAT3 was elevated after 30 minutes of treatment with recombinant IL-6 (10 ng/ml) and sIL-6R (25 ng/ml), and that addition of neutralizing anti-IL-6 mAb blocked this activation. Likewise, conditioned media (CM) from NBT2 cells co-cultured with macrophages activated STAT3; however, this effect could not be blocked by anti-IL-6 mAb, suggesting that other secreted factors can contribute to STAT3 activation (Figure 4D). Experiments conducted using the human CHLA-255 cell line replicated our findings in the murine system (Figure 4D), showing that IL-6 is not the only factor involved in STAT3 activation in NBL. Correlation analysis of the expression profile data also did not identify any single cytokine activator of STAT3 that was highly correlated (r >0.75) with CD163 expression (Supplementary Table 1). These data suggest that additional activators of STAT3 phosphorylation play a role in the NBL-TAM cell interactions, and that therapeutic interventions should focus on events downstream of the ligand-receptor systems known to activate STAT3.

### STAT3 inhibition decreases macrophage-induced expression of MYC in NBL cells and reverses the proliferative effects of macrophages

To assess the importance of STAT3 signaling on macrophage-induced NBL cell proliferation, we used two potent STAT3 inhibitors, AZD1480 and ruxolitinib, to block STAT3 phosphorylation via inhibition of the Janus kinase 2 (JAK2) kinase, which is downstream of IL-6R and upstream of STAT3. Both ruxolitinib and AZD1480 significantly reduced NBT2 proliferation by an average of 40% (p<0.001, and p<0.05, respectively; Figure 5A). However, proliferation of NBT2 co-cultured with macrophages was only significantly reduced by ruxolitinib (p<0.001). Ruxolitinib was also more effective than AZD1480 at blocking the macrophage-dependent STAT3 phosphorylation in NBT2 cells (Figure 5B). Inhibition of pSTAT3 also led to a partial decrease in MYC protein levels, suggesting that the regulation of MYC expression by macrophages can have an IL-6-independent component, but requires STAT3. Ruxolitinib was also effective at blocking the macrophage-dependent STAT3 phosphorylation and MYC up-regulation in *MYCN* non-amplified human neuroblastoma cells (Figure 5C).

*In vivo*, ruxolitinib was effective at reducing pSTAT3 levels in tumor lysates obtained from treated NB-Tag mice or NOD scid gamma (NSG) mice bearing subcutaneous tumors compared to tumor lysates from untreated animals (Figure 5D). We next assessed the effect of ruxolitinib on growth of subcutaneously implanted NBT2 cell lines with and without co-injection of macrophages that were pre-conditioned for 24 hours with tumor cells *ex vivo* (Figure 5E). The proliferative advantage provided by macrophages to NBT2 cells in animals was effectively inhibited by ruxolitinib (ANOVA p = NS between NBT2 vs. NBT2+Macrophages) compared to untreated animals (ANOVA p < 0.001 between NBT2 vs. NBT2+Macrophages) (Figure 5E).

## DISCUSSION

TAMs are known to promote tumor progression via multiple mechanisms regulating tumor cell growth, survival, invasion, metastasis, angiogenesis, inflammation, and immune regulation [2, 4, 24]. The presence of TAMs has been described in many adult and childhood malignancies, including NBL [6, 25, 26]. Previous work attributed the monocyte/macrophage-induced proliferative effect in NBL to activation of the IL-6 pathway [27], In this study, we took advantage of the relatively recent development of the NB-Tag mouse model, which we characterized as a model of NBL lacking *MYCN* amplification, to assess the development of the NBL TME and the role of TAMs. We demonstrate that NBL cells recruit macrophages early in their development by expressing CCL2. TAMs and circulating monocytes in turn produce high levels of IL-6, contributing towards establishment or maintenance of an inflammation-rich TME. Findings of increased IL-6 levels in NB-Tag mice are consistent with the elevated IL-6 levels found in bone marrow aspirates and sera of children with metastatic and relapsed NBL [11, 28]. In human and murine *in vitro* co-culture studies, we demonstrated that NBL-TAM interactions led to increased IL-6 levels in the media, increased tumor cell proliferation and decreased apoptosis. However, we found that genetic ablation of the *IL6* gene in NB-Tag mice led to similar tumor growth pattern as in their IL-6-producing counterpart, and macrophages isolated from *IL6* knockout mice were just as capable of increasing NBL proliferation *in vitro*. These findings indicate that TAMs mediate activation of STAT3 and promotion of NBL growth not only in an IL-6-dependent manner [11] but also in a redundant, IL-6-independent manner.

The trimeric interaction of IL-6 with soluble IL-6R and the common gp130 subunit is known to activate STAT3 and promote tumor growth [7, 16, 17, 29, 30]. However, STAT3 activation can also occur through the binding of other members of the IL-6 family of cytokines (nerve trophic factor, leukemia inhibitory factor, oncostatin M, IL-11, cardiotrophin-1) to gp130, or via ligation of growth factors such as epidermal growth factor, platelet-derived growth factor, or oncogenic proteins including Src and Ras to their respective receptors [31]. Persistent activation of STAT3 can in turn function as a master regulator of molecular and biological events and promote growth and inhibit apoptosis [20, 30, 32]. The findings herein show via gene expression profiling and protein analysis that macrophages activate STAT3 in NBL cells and demonstrate for the first time that this macrophage-induced activation of STAT3 can result in up-regulation of MYC in NBL cells. STAT3 has been previously shown to increase MYC expression in other experimental systems. Forced expression of activated STAT3 in fibroblast cell lines resulted in 3-6 fold up-regulation of *MYC*, *CCND1* (coding for cyclin D1) and *BCL2L1* (BCL-XL) mRNA, and permitted tumor formation in nude mice [30]. Activation of STAT3 protein in the pro-B hematopoietic cell line BAF-B03 has also been shown to induce binding of STAT3 directly to the *E2F* site of the *MYC* promoter, increasing *MYC* mRNA expression 2-3 fold [33]. Our results show a similar 2-3 fold increase in the level of MYC protein in NBL cells co-cultured with macrophages. Our observation that this increase occurs when using *IL6*^KO^ macrophages *in vitro* and in NB-Tag/*IL6*^KO^ tumors *in vivo* suggests that there are redundant pathways in NBL through which TAMs activate STAT3 and up-regulate MYC.

The recent discovery of MYC (c-MYC) protein expression in ∼15-20% of high-risk NBL patients has uncovered the importance of this oncogene in NBL [23]. The MYC-family (MYCN or MYC) protein-expressing subset of NBL was shown to be highly aggressive and to confer poor prognosis [23]. Our previous study in children with high-risk NBL lacking *MYCN* amplification showed that expression of a 14-gene signature that included *CD14* (monocyte/macrophage marker) and *IL6R* could identify children with extremely poor survival [6]. In addition, *IL6R* gene expression showed a positive correlation with expression of NTRK2, a known marker of poor prognosis in NBL. In this study, using a large cohort of 249 primary NBL samples, we identified a significant positive correlation between expression of TAM markers (*CCL2*, *CD14*), and *MYC*, an observation that supports our *in vitro* and *in vivo* findings. It is also worth noting that while expression of CD14 is highly correlated with MYC expression, few MYCN amplified tumors express high levels of CD14 (Figure 2B). It is possible that this small subset of MYCN amplified tumors also rely on TAMs for growth or represent expression of CD14 on macrophage subsets that are present within the necrotic portions of these tumors. The *in vitro* and *in vivo* inhibition of JAK1/2 by ruxolitinib abolished the macrophage-induced tumor proliferative effects and decreased MYC expression. It has previously been shown that inhibition of the JAK-STAT pathway using the tool compound AZD1480 could limit the expansion of the human NBL cell lines SMS-KCNR and SY5Y growing subcutaneously in immune compromised nude mice [34]. However, development of AZD1480 for clinical use has been discontinued by the manufacturer. Therefore, we examined a clinically admissible JAK-STAT inhibitor, ruxolitinib, and in NSG mice demonstrate for the first time that ruxolitinib can be used to slow the growth of NBL.

In summary, the characterization of the TME in the NB-Tag model and functional analyses of NBL-TAM interactions provided here reveal new facets of the biology of NBL lacking *MYCN* amplification. We demonstrate that NBL-TAM interactions lead to MYC protein up-regulation through the STAT3 pathway, and while IL-6 levels are increased during this interaction, IL-6 is not essential and is redundant with alternative mechanisms of STAT3 activation. Thus, pharmacological inhibition of IL-6 alone or of alternative, competing ligands for the common gp130 subunit of IL-6R may be insufficient to block growth and proliferation. Our study indicates that targeting STAT3 or events downstream of its activating ligand-receptor systems such as the JAK pathway may be a promising approach to block NBL-TAM interactions and improve outcome in children with high-risk NBL. Our observation that TAMs can up-regulate MYC protein expression in NBL cells may explain, at least in part, why TAMs are strongly associated with poor survival in NBLs that lack *MYCN* amplification.

## MATERIALS AND METHODS

### Neuroblastoma models

All experimental procedures were approved by the Children’s Hospital Los Angeles Institutional Animal Care and Use Committee. Spontaneously arising NB-Tag tumors and tumors formed by subcutaneous injection of the NBT2 NBL cell line were monitored by magnetic resonance imaging (MRI) on a Biospin preclinical MRI platform after injection with 30 μL of Magnevist (Bayer Pharmaceuticals, Leverkusen, Germany). Images were analyzed using the ImageJ program (National Institutes of Health). Tumors were measured in two dimensions and tumor volume was estimated with following the formula: volume = 0.5 x length x width^2^. *NB-Tag murine model*: Transgenic mice carrying the SV40 large T-antigen gene (NB-Tag) have been described earlier [21]. *MYCN* gene amplification status in the NB-Tag tumors was determined by comparative genomic hybridization assay (CGH) using Nimblegen murine 385K aCGH arrays and comparing tumor DNA against germline DNA per the manufacturer's instructions (MOgene, St. Louis, MO). Double transgenic NB-Tag/*IL6*^KO^ mice were generated by crossing NB-Tag male mice with *IL6* knockout female mice (B B6.129S2-*Il6*^*tm1Kopf*^/J, Jackson Laboratory, Bar Harbor, Maine). *Subcutaneous Murine Model*: The murine NBT2 NBL cell line was established from the adrenal tumor of a 16-week-old NB-Tag mouse. Its NBL identity was validated by RT-PCR for the TH gene, while its tumorigenic character was validated by subcutaneous tumorigenicity studies in NSG and syngeneic C57/BL6 mice. Subcutaneous tumors were generated by injecting 1x10^6^ cells in the shoulders of NSG mice and measurements were performed with calipers in two dimensions. *Human NBL Cell lines:* The human NBL cell lines used in this study (CHLA-255, LAN-6 and LAN-5) have been previously described [35]. *Murine Tumor-Macrophage Co-Injection Model:* To evaluate the growth promoting effect of macrophages on NBT2 cells *in vivo*, 8-week old NSG mice were inoculated subcutaneously with 1x10^6^ NBT2 cells. One shoulder of the mice was co-injected with equal amount of NBT2 cells and macrophages while the other shoulder was injected with NBT2 cells only. Macrophages were isolated from peritoneum of WT mice as described below, conditioned for 36 hours in the lower chambers of transwell plates while the upper chambers contained NBT2 cells. The conditioned F4/80^+^ macrophages were co-injected with equal numbers of naïve NBT2 cells (*i.e*., NBT2 cells that had not been used to condition macrophages). *Human cell lines*: All cells lines were established by researchers at our institute and maintained in a humidified incubator with 5% CO_2_ at 37°C in the medium in which they were established. CHLA-79, CHLA-172, and CHLA-255 were maintained in Iscove’s Modified Dulbecco’s Medium (IMDM) supplemented with 10% fetal bovine serum (FBS). LAN-5 and LAN-6 cells were maintained in RPMI with 10% FBS. Cell lines were regularly tested for mycoplasma using the MycoAlert kit (Lonza, Allendale, NJ) and for correct identity using the AmpFLSTR Identifiler PCR Amplification Kit (Thermo Fisher Scientific, Waltham, MA) and were tested at the completion of the study.

### Macrophage isolation and co-culture experiments

Mouse macrophages were isolated from peritoneal cavity cell populations by magnetic cell sorting (MACS) positive selection with F4/80-APC antibody (Ab) and anti-APC Ab-coated microbeads (Miltenyi Biotec, Auburn, CA). Isolation of peritoneal cavity cells was conducted as previously described [36], but without using thioglycate to increase recruitment. Briefly, peritoneal washings were obtained from 10-12 week-old mice using ice-cold MACS buffer (0.5% BSA and 2 mM EDTA in PBS) and cells were labeled with the anti-F4/80-APC Ab. These cells were then incubated with anti-APC microbeads and passed through a MACS separator column as per the manufacturer’s protocol. The phenotype and purity of macrophages were confirmed using flow cytometry and mAbs F4/80-APC and anti-CD11b-PE (clone M1/70, eBioscience Inc, San Diego, CA). F4/80^+^ cells isolated as above were co-cultured with NBT2 (1x10^5^ cells/well; 1:1 ratio) cells in 12-well dishes in IMDM plus 2% FBS for 36 hours. For transwell co-cultures, F4/80^+^ cells were plated in the transwell inserts (0.4 μm, Costar) at a density of 1x10^5^ cells per insert with the NBT2 cells at the bottom of the 12-well plates. For determining proliferation, cells were pulsed with BrdU for 45 minutes before being harvested with Accumax (Millipore, Hayward, CA), followed by staining with anti-CD45-APC mAb, fixation, permeabilization, and staining with anti-BrdU-FITC and 7-Aminoactinomycin D (7AAD) counterstain (BD Biosciences, San Jose, CA) according to the manufacturer’s instructions. NBL cells in S-phase of the cell cycle were identified as BrdU positive events after excluding macrophages according to their expression of CD45 using FCS Express v3 software (De Novo Software, Los Angeles, CA). Cells in direct co-cuture were separated into NBL and immune cell fractions for RNA and protein extraction using CD45-APC staining and MACS (Miltenyi Biotec).

For experiments using human cells, human peripheral blood mononuclear cells (PBMC) were freshly isolated by Ficoll-Paque gradient centrifugation from discarded leukocyte filters obtained during platelet collection from healthy adults at the Children’s Hospital Los Angeles Blood Collection Center. Monocytes were selected using the Monocyte Isolation Kit II (Miltenyi Biotec), and differentiated to M2-like macrophages by culturing in IMDM containing 10% FBS supplemented with 10 ng/ml M-CSF-1 for 7 days as described earlier [37]. These macrophages were co-cultured with human NBL cell lines in IMDM containing 3% FBS for 48 hours. Cell proliferation was determined by BrdU and flow cytometry as described above.

### RNA and protein analyses

Total RNA was isolated using the STAT-60 RNA reagent and cleaned by passing through RNeasy columns (Qiagen, Valencia, CA). For RT-PCR assays, clean RNA was used as template for cDNA transcription using SuperScript® III Reverse Transcriptase (Life Technologies, Grand Island, NY), and this was used as template for real-time PCR using mouse immune Taqman Low Density Arrays (Life Technologies, Carlsbad, CA) or pre-designed gene specific primer probe sets. Total RNA was also used to assess gene expression with the Nanostring nCounter Mouse Inflammation Kit (Nanostring Technologies, Seattle, WA). *Immunohistochemistry analyses*: Adrenal gland or tumor from 4-, 8-, 12- and 16-week-old WT and NB-Tag mice were fixed and embedded with 4% paraformaldehyde and paraffin wax. Macrophages, TH levels and pSTAT3 were detected using rat anti-mouse F4/80 (Invitrogen, Carlsbad, CA: MF48000), unlabelled rabbit anti-mouse tyrosine hydroxylase polyclonal Ab (Abcam, Cambridge, UK: ab112) and rabbit unlabeled rabbit anti-mouse pSTAT3 (Tyr705) mAb (clone D3A7, Cell Signaling Technology, Danvers, MA), respectively. Serum IL-6 and CCL2 levels were quantified for animals of different ages using the Luminex assay and the MILLIPLEX MAP Mouse Cytokine/Chemokine - Premixed 32 Plex kit (Millipore, Temecula, CA). IL-6 released into culture media during co-culture experiments was quantified using the DuoSet mouse IL-6 Elisa kit (R&D systems, Minneapolis, MN) according to the manufacturer’s protocols. *Immunoblotting*: Blots were probed with rabbit polyclonal Ab against pSTAT3 (Tyr705) (pAb #9131) and mAb against STAT3 (clone 79D7) (Cell Signaling Technology, Danvers, MA) or mAb against c-MYC (clone Y69) (Abcam, Cambridge, UK), and detected using donkey anti-rabbit IRDye fluorescently-labeled secondary Ab (LI-COR Biosciences, Lincoln, NE). The detection and quantification were conducted using the Odyssey Infrared Imaging Systems (LI-COR Biosciences).

### Flow cytometry analyses

Mouse tumors were excised and dissociated using a gentleMACS^™^ dissociator (Miltenyi Biotec) according to the manufacturer’s protocol. Peripheral blood was collected in tubes containing 2% EDTA solution (Sigma-Aldrich, St Louis, MO) to obtain single cell suspensions. Brefeldin A, a protein transport inhibitor (BD Biosciences, San Jose, CA), was used in all pre-fixation/permeabilization buffers. Cells were surface-stained with a pre-mixed fluorescence-conjugated mAb cocktail or isotype controls for 45 minutes at 4°C in the dark. The cocktails were prepared in 5 different combinations [(1) CD45, CD11b, Ly6G; (2) CD45, CD11b, F4/80; (3) CD45, CD11b, B220; (4) CD45, CD11b, CD8a; (5) CD45, CD8a, CD4]. Murine blood samples (25-35 μl per tube) were stained with the same Ab cocktails, and RBCs were removed after surface staining using BD Pharm Lyse (BD Biosciences, San Jose, CA) according to manufacturer’s protocols. DAPI (0.5 ng/ml final concentration) was added to all non-fixed samples. For intracellular staining, surface-stained cells were fixed with 0.128% formaldehyde for 10 minutes at 37°C, washed with staining buffer, and permeabilized by washing twice with BD Perm/Wash buffer (BD Biosciences). Permeabilized cells were incubated with rat anti-mouse IL-6-FITC mAb (clone MP5-20F3) (eBioscience) for 45 minutes at 4°C, subsequently washed twice with BD Perm/Wash and analyzed by flow cytometry. Data were acquired on a four-laser LSR-II flow cytometer (BD Biosciences) using a UV laser to excite DAPI. Analysis was performed using BD FACSDiva software v. 6.0 and FlowJo 10.0.6 (Tree Star, Inc, Ashland, OR). mAbs and fluorochromes included anti-mouse CD45-APC-Cyanine7, anti-mouse CD4-FITC, anti-mouse F4/80-PerCp-Cy5.5, anti-mouse F4/80-APC, anti-mouse Ly6G-APC (Biolegend, San Diego, CA) as well as anti-mouse CD4-APC-H7, anti-mouse CD19-PerCP-Cyanine5.5, anti-mouse CD11b-PE, anti-mouse B220-PE (BD Biosciences) and corresponding isotype controls.

### STAT3 inhibition experiments

NBT2 cells were cultured with and without macrophages in the presence of the STAT3 inhibitor AZD1480 or ruxolitinib (Selleckchem, Houston, TX). At different time points, NBT2 cells were collected from the bottom chambers of transwells and lysed in RIPA buffer supplemented with protease and phosphatase inhibitor cocktail (Roche, Indianapolis, IN). The lysates were centrifuged at 13000 x g for 20 min at 4°C, and the supernatants were used to assess STAT3 phosphorylation by immunoblotting. To assess the effectiveness of ruxolitinib *in vivo*, NB-Tag mice (14 weeks of age) or NSG mice with palpable NBT2 subcutaneous tumor were treated with ruxolitinib twice daily for one week. A stock solution of ruxolitinib was prepared in dimethyl sulphoxide (DMSO) and suspended in vehicle (Saline) prior to treatment. Mice were treated twice daily by oral gavage with vehicle or ruxolitinib (60 mg/kg). Tumors were dissected 1 hour after the last dose of ruxolitinib to assess the status of pSTAT3. Tumor lysates were treated as above for immunoblotting.

The subcutaneous tumor-macrophage co-injection model described above was used to test the efficacy of ruxolitinib at inhibiting macrophage-induced tumor growth *in vivo*. Mice were randomized into two different treatment arms. Mice were treated twice daily by oral gavage with vehicle or ruxolitinib (60 mg/kg) from the 2^nd^ day after tumor injection and then continuously for 3 weeks. The primary endpoint was achievement of 500 mm^3^ tumor volume.

### Statistics

Nanostring’s pre-built nCounter® Mouse Inflammation v1 was used to identify differentially expressed genes between macrophages and NBL cell lines in co-culture experiments. Data were normalized per the manufacturer’s recommendations and analyzed using nSolver software (Nanostring Technologies, Seattle, WA). Gene expression profiling of NB-Tag tumor samples was performed on GeneChip® Mouse Genome 430 2.0 Array (Affymetrix, Santa Clara, CA) per manufacturer recommendations. GeneChip® Human Genome U133A Array data from GSE3446 representing human neuroblastomas and GSE1133 representing various normal human tissues were used for cross-species analysis. Background correction and normalization were performed in mouse and human datasets using the Robust Multi-array Average (RMA) algorithm as implemented in the APT tools (Affymetrix, Carlsbad, CA), average gene expressions were calculated, and both data were converted to z scores. Genes found to be highly variable (coefficient of variation greater than 0.2) in the human neuroblastoma dataset were selected for and analyzed for their first two principal components.

All statistical analyses were performed using R version 3 (The R Project for Statistical Computing). Differences in means were determined with the Student’s t-test, not assuming equal variances; the Wilcoxon rank test was substituted where indicated for non-normal data. *In vivo* co-culture growth analysis was performed using linear regression with group as the independent variable. A p-value of 0.05 was used as the cutoff for statistical significance.

## SUPPLEMENTARY MATERIALS FIGURES AND TABLE




