# Supplementary material for: Tumor-associated macrophages promote neuroblastoma via STAT3 phosphorylation and up-regulation of c-MYC
Source: Oncotarget. 2017 Sep 16;8(53):91516–29. doi: 10.18632/oncotarget.21066 (PMC5710942; doi:10.18632/oncotarget.21066)
Supplement: Supplementary file 1 [file oncotarget-08-91516-s001.pdf]

Tumor-associated macrophages promote neuroblastoma via STAT3 phosphorylation and up-regulation of c-MYC

SUPPLEMENTARY MATERIALS

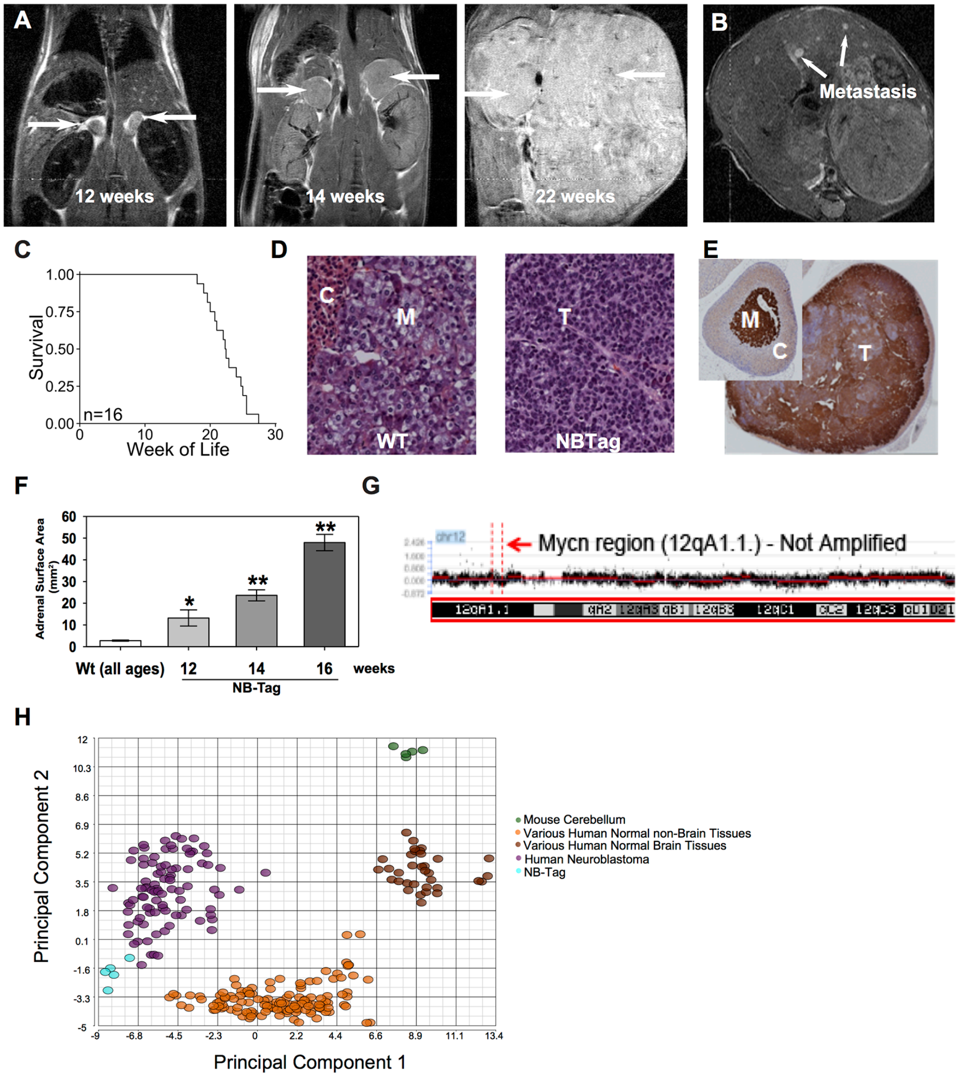

Supplementary Figure 1: NB-Tag represents an immunocompetent spontaneous murine model of human high-risk MYCN non-amplified neuroblastomas. **(A)** Representative MRI images of tumor growth in the bilateral adrenal glands (white arrows) of NB-Tag mice at different ages; **(B)** MRI image of liver metastasis in NB-Tag mice, typically evident around 22 weeks of age; **(C)** Survival analysis of NB-Tag mice (n=16) shows 100% penetrance and lethality of the disease; **(D)** H&E analysis of formalin-fixed, paraffin-embedded sections of normal adrenal gland (left photomicrograph; C, Cortex; M, Medulla) and of tumor (right photomicrograph; T, tumor) showing undifferentiated small blue round tumor cells with high mitosis-karyorrhexis index; **(E)** Immunohistochemical (IHC) staining for tyrosine hydroxylase (TH) of 12-week-old NB-Tag tumor compared to normal adrenal gland (magnification ×200); **(F)** Mean (+SD) surface area of adrenal glands in WT mice (n = 7) and NB-Tag tumors at 12, 14, and 16 weeks (n = 5-6 mice per group) as assessed from MRI images (* $p < 0.05$;, ** $p < 0.005$); **(G)** Comparative Genomic Hybridization plot of the chromosome 12 region, which harbors the mouse *MYCN* gene (red dashed lines), for a NB-Tag tumor. The Y-axis indicates the log2 ratio for each probe set (log2 ratio of 0 indicates normal copy number), a positive log2 ratio indicates amplification, while a negative log2 ratio indicates deletion; **(H)** Scatter plot of the first two principal components of z-score normalized gene expression data from microarray studies of NB-Tag tumors (cyanide; n=5 unique tumors), mouse normal cerebellum (green; n=5 unique cerebella), human primary neuroblastoma tumors (purple; GSE3446), and various normal human brain (brown) and non-brain (orange) tissues (GSE1133).

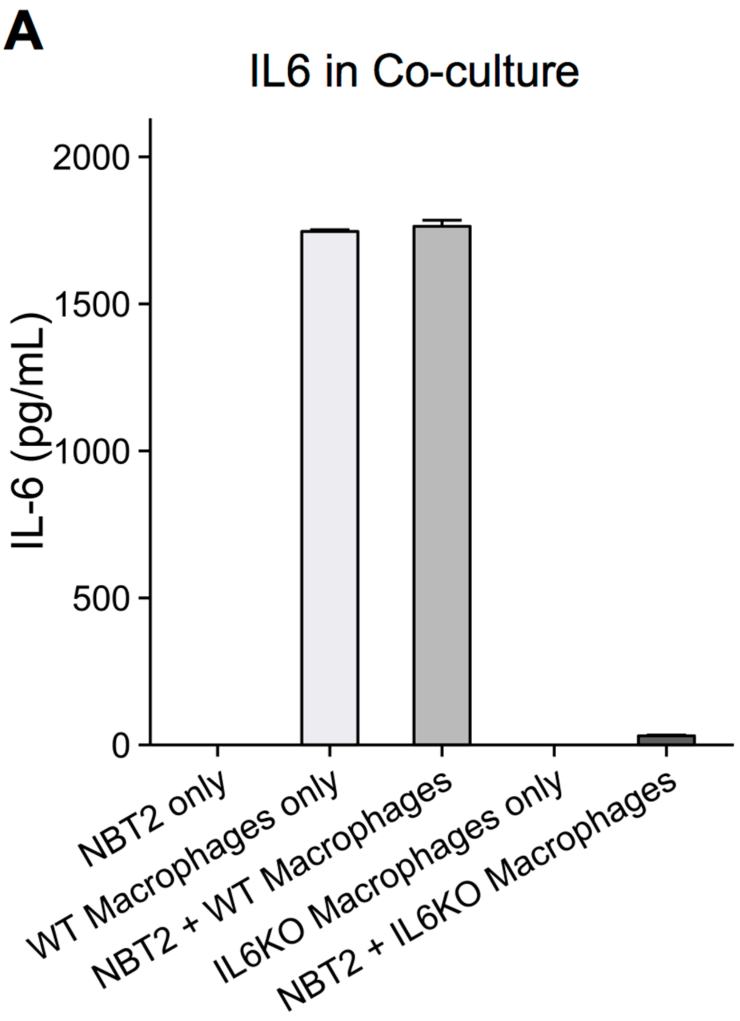

Supplementary Figure 2: (A) Mean (+SD) IL-6 concentration in media from cultures of NBT2 cells, cultures macrophages from *IL6* WT and KO animals, and their co-cultures. (n = 3 independent experiments per group), as assessed by Luminex.

Supplementary Table 1: List of genes from the TARGET microarray data (n=249 samples) that have correlation (spearman correlation) value greater than 0.75 with CD163 expression

See Supplementary File 1
